# Nurse Managers' Leadership Styles in Finland

**DOI:** 10.1155/2012/605379

**Published:** 2012-09-12

**Authors:** Soili Vesterinen, Marjo Suhonen, Arja Isola, Leena Paasivaara

**Affiliations:** ^1^Lapland Central Hospital, Lapland Hospital District, Box 8041, 96101 Rovaniemi, Finland; ^2^Department of Health Sciences, University of Oulu, Box 5000, 90014 Oulu, Finland

## Abstract

Nurse managers who can observe their own behaviour and its effects on employees can adjust to a better leadership style. The intention of this study was to explore nurses' and supervisors' perceptions of nurse managers' leadership styles. Open-ended interviews were conducted with 11 nurses and 10 superiors. The data were analysed by content analysis. In the study, six leadership styles were identified: visionary, coaching, affiliate, democratic, commanding, and isolating. Job satisfaction and commitment as well as operation and development work, cooperation, and organizational climate in the work unit were the factors, affected by leadership styles. The nurse managers should consider their leadership style from the point of view of employees, situation factors, and goals of the organization. Leadership styles where employees are seen in a participatory role have become more common.

## 1. Introduction

Health care is changing dynamically in the 2010s. The economic recession and problems with recruiting professionals [1, 2], staff retention [3], creating healthy work environments [4, 5], and a growing demand for customer orientation [6] pose challenges for nurse managers' work. More expertise in management is needed to respond to these issues. 

One essential area of nurse manager's management skills is the use of different leadership styles [7]. Leadership styles can be seen as different combinations of tasks and transaction behaviours that influence people in achieving goals [8]. Earlier studies indicate that nurse manager's effective leadership style is affiliated to staff retention [5], work unit climate [4], nurses' job satisfaction [9, 10], nurses' commitment [11], and patient satisfaction [12]. 

Transformational leadership style [5, 6, 13, 14] and transactional leadership [7] help to respond to these issues. Transformational leadership refers to the leader's skills to influence others towards achieving goals by changing the followers' beliefs, values, and needs [7]. Transactional leadership complements and enhances the effects of transformational leadership outcomes [15]. 

There are certain skills required from nurse managers so as to be able to use these effective leadership styles. The skills include the ability to create an organization culture that combines high-quality health care and patient/employee safety and highly developed collaborative and team-building skills [1]. Nurse managers also need to have the readiness to observe their own behaviour [16] and its effects on the work unit; as a result, employees can adjust to a better leadership style. These kinds of skills are related to manager's emotional intelligence (EI). 

EI is an ability to lead ourselves and our relationships effectively [17]. It has been defined as the ability to observe one's own and others' feelings and emotions, to discriminate among them and to use this information to direct one's thinking and actions [18]. EI is composed of personal competence and social competence. Self-awareness and self-management are reflections of personal competence, influencing the way the leader manages him/herself. Social awareness and relationship management reflect social competence, which affects how the leader manages relationships with others [17]. 

Nurse managers with that skill can easily form relationships with others, read employees' feelings and responses accurately, and lead successfully [19–21]. Emotionally intelligent leaders' behaviour also stimulates the creativity of their employees [22]. Goleman et al. [23] have identified visionary, coaching, affiliate, and democratic styles as resonant, and pacesetting and commanding styles as dissonant leadership styles. Most leaders use both resonant and dissonant leadership styles. The leadership styles of Goleman et al. are applied as the basis of this study because earlier studies refer to the significance of these styles, especially that of EI in manager's work. In addition, these leadership styles are one way of aiming to carry out transformational leadership. Especially visionary, coaching, affiliate, and democratic styles include elements that promote transformational leadership. Such elements are for example the leader being visionary and empowering staff [4]. 

This paper focuses on Finnish nurse managers' leadership styles. The Finnish health care system is a strong institution where health care services are offered to all citizens and funded by taxes [24]. It has widely recognized that health care services in Finland are of high-quality Despite recent concerns about equity issues, Finns are in general very satisfied with their health care services. [25]. Consequently it is important to explore nurse managers' leadership styles especially in this context. 

## 2. Materials and Methods

### 2.1. Aim of the Study

The intention of this study was to explore nurses' and supervisors' perceptions of nurse leaders' leadership styles. The research questions were as follows: what kind of leadership styles do nurse managers use and what are the factors affected by their leadership styles. 

### 2.2. Participants

To achieve the aim of this study data were collected through open interviews. The majority of Finnish nurse managers, nurses, and supervisors work in hospitals or long-term facilities. Selection of participants was performed in convenience sampling [26]. Participants were selected paying attention to the fact they were of different ages, working in different wards and units (e.g., psychiatry, internal diseases, gerontology) in either hospitals or long-term facilities, and had worked with more than one nurse manager. 

The researcher contacted the participants and asked whether they were interested in taking part in the study. The participants were informed about the aim of the study. Participation was voluntary. Prior to the interviews each participant signed a form where they gave their consent to participate in the study. A total of 11 nurses and 10 supervisors, 20 women and one man, from eight Finnish hospitals and five long-term care facilities participated in the study. The age of the nurses varied between 30 and 53 and their experience in health care between 7 and 25 years. The age of the supervisors varied between 38 and 59 and their experience as supervisors between 5 and 21 years. Both nurses and supervisors had worked with many nurse leaders and they were interviewed about nurse managers in general. They thus had experience of different nurse managers on different wards and they were able to describe leadership styles from various aspects. 

### 2.3. Data Collection and Analysis

Semistructured interviews were used to gather data on the perceptions of nurse managers' leadership styles and factors affected by leadership styles. Interviews were usually carried out in the office in the participants' workplace. All interviews were recorded with individual consent. Participants were initially asked to describe their work and earlier study and work history. They were subsequently asked about their perception of leadership styles and asked to describe the leadership styles used by their nurse managers. After that they were asked about factors affected by leadership styles. Each interview was approached individually, guided by participants' responses. The interview sessions lasted between 30 and 85 minutes. Every interview was transcribed word for word from the recordings. Interviewing was continued until saturation of the data was achieved [27]. 

Because nurses and supervisors might have differed in their perceptions of leadership styles, the data were first analysed separately in two separate groups, following the same process for each group. Content analysis was chosen because it is a research method for making valid inferences from data to the contexts of their use [28]. 

The interview texts were read through multiple times, based on the author's empirical and theoretical preunderstanding of the professional area of the participating nurses and nurse managers. A structured categorization matrix of leadership styles was developed based on the primal leadership model [23] and research of Vesterinen et al. [29]. When using a structured matrix of analysis, an item of the data that does not fit the categorization frame is used to create its own concept, based on the principles of inductive content analysis [30]. When both the data of nurses and superiors were analysed, the results were compared. The categories and subthemes were congruent and therefore the results are presented together, albeit paying attention to differences and similarities of the perceptions of nurses and superiors. 

The data analysis of the factors affected by leadership styles was inductive. All the data of nurses and supervisors were analysed together. This process included open coding, creating categories, and abstraction. A classification framework of the factors was formed inductively by defining categories and sub-themes. The criteria for allocating a unit to a category were formed by asking questions if the unit was suitable to the category. The sub-themes were named using descriptive concepts and classified as “belonging” to a particular category. After that, the categories were given names [31]. 

### 2.4. Trustworthiness

The trustworthiness of this study has been ensured by confirming truth value, consistency, neutrality, and transferability of this study [32]. 

When considering this study from the viewpoint of trustworthiness, there are some threats that should be taken into consideration. The researcher collected the data and performed the analysis alone and the interpretation could have been affected by her professional history [33]. With interviews there is a risk that respondents try to please the interviewer by reporting things they assume s/he wants to hear. The researcher confirmed the truth value of the study by selecting participants in convenience sampling. The respondents' age distribution was wide and they worked in different units. Their perspectives and descriptions were broad and gave a diverse picture.

The truth value of this study was also confirmed by analysing data as they emerged based on the interviews. To ensure the trustworthiness of the study quotes from interviews are included in the results. 

In view of consistency, the research process is described so that it can be repeated if necessary. This gives a possibility to understand the limitations of the process of data collection and analysis. To ensure neutrality in this study, interpretations were based on original data. This is confirmed by citations from the interview data.

In this study the sample was small, consisting of Finnish nurses and supervisors, and the results only reflect their perceptions of leadership styles. As a result, transferability of results is limited. However, when considering the main objective in this study, it was not transferability of research results, but it was to enhance understanding of leadership styles and use it for future studies. 

### 2.5. Ethical Considerations

The data for this study were collected following approval from the administrations of the organizations. All participants were informed of the purpose of the study. They were told that their participation was voluntary and would be treated with confidentiality. Participants were asked to sign a form where they gave their consent to take part in the study. 

## 3. Results and Discussion 

### 3.1. Results

**Figure 1:**
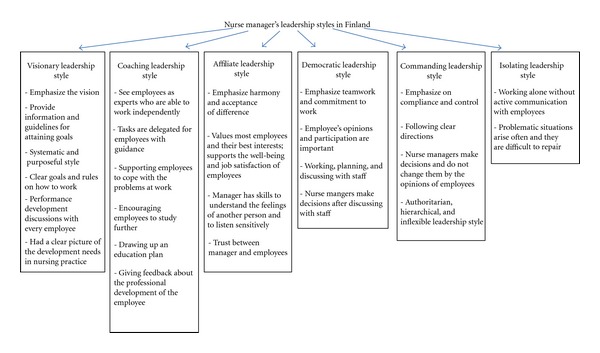
Nurse managers' leadership styles in Finland. Summary of findings of the study.

Data analysis identified visionary, coaching, affiliate, democratic, commanding, and isolating leadership styles (Figure 1). Job satisfaction and commitment as well as operation and development work, cooperation and organizational climate in the work unit were the factors affected by leadership styles.

#### 3.1.1. Leadership Styles

**Visionary Leadership Style**

Supervisors were of the opinion that today, nurse managers use a more visionary leadership style than previously. In the past, many organizations lacked a vision of their own and had fewer possibilities to engage in development for the future. Even now, the skills of nurse managers to lead visionary development work varied.

Both nurses and supervisors reported that it was characteristic of the visionary nurse manager to emphasize and discuss the vision and provide information to employees. When establishing their vision, some nurse managers provided guidelines for attaining the work unit's goals. These nurse managers had a systematic and purposeful leadership style, based on the knowledge of nursing science and practice. They generally worked in organizations with strategies and vision. They had clear goals and rules on how to work. Nurse managers had so-called performance development discussions with every employee once a year. During the discussion, the nurse manager explained and revised the goals and discussed the purpose of the employee's work together with each employee. At the same time, they agreed on the goals of the employee for the next year. Visionary nurse managers were described as being assertive and persistent in their attempts to get the work units to achieve their goals. Nurse managers with more recent education were better equipped to search for information than nurse managers with older education. In addition, they often had a clear picture of the development needs in nursing practice. 

Supervisors said that sometimes the fact that the organization did not have visions or direction for the future was an obstacle to a visionary leadership style. This was emphasized in cases where changes were introduced to the organization. Some nurse managers worked more on the basis of operation up until the present. The managers were guided by various situations and there were no plans for the future.

*“This manager had visions and we had long-term plans, but these plans often changed.”*

Nurses emphasized the importance of making the vision understandable by giving information about current issues of the work unit. Nurse manager's skills to provide information objectively and positively influenced the way the personnel reacted to topical issues. It was also important to explain the motivation behind decisions.

**Coaching Leadership Style**

Nurses as well as supervisors felt that nurse managers with a coaching leadership style took into consideration both the professional development of the employees and delegation of work. The employees had resources and were seen as experts and the nurse manager delegated tasks to them. The skills of employees to work independently varied. Some employees needed more coaching while others were satisfied with using their own professional skills independently.

The success of delegation was affected by common instructions. They guided employees so that every employee knew his/her tasks. The employees worked and made decisions independently within the bounds agreed. The nurse managers had a significant role in supporting the employees to cope with the problems at work. They were also responsible for coordinating and organizing work in the unit as a whole.

*“A nurse manager draws plans for nursing practice so that there are these areas of responsibility and everybody knows what is their area and they answer for that.”*

The nurse manager paid attention to employees' professional skills and encouraged them to study further. Both personnel's competence and leaders' skills to lead influenced the development work in the unit. It was useful to clarify what kind of needs the work unit and employees had for additional education and to draw up an education plan. This plan was a meaningful basis to guide the employees to necessary training. It was each employee's duty to share the new knowledge with other employees. The nurse manager encouraged the employees to collect information without prompting and to think independently. S/he also gave feedback about the professional development of the employee.

**Affiliate Leadership Style**

Nurses as well as supervisors described an affiliate leadership style. Nurse managers with affiliate leadership style emphasized harmony and acceptance of difference. The employees and their best interests were the most important value to the nurse manager. They knew the rules and guidelines of the organization, but they considered the hopes and needs of employees in a flexible manner. The nurse manager had skills to understand the feelings of another person and supported him/her by listening sensitively. Both s/he and his/her personnel trusted each other. Nurses reported that this encouraged the employees to discuss their personal concerns with the nurse manager.

*“The way to act, pay attention to the employee, do you listen to her or not, that is the basic question.”*

On the other hand, supervisors reported that leading could be too solicitous, in a completely motherly way. The basis of the leadership style could be supporting the well-being and job satisfaction of the employees; this might be more important than the development of nursing practise. The purpose—a harmonious atmosphere without conflicts—can be an obstacle to planned changes.

*“When there are big changes in the work unit, nurse manager is present to the employees and listen [sic] to them. She tries to support and say [sic]: there is no problem and we manage of this.”*

*“When a new employee begins to work, she leads in [sic] more paternalistic way and takes care of them all the time.”*

Both nurses and supervisors deemed it important that the nurse manager respects differences and personal characteristics of the employees, not forgetting employees' equality. A nurse manager who respects and accepts the employees as individuals was easy to approach. On the other hand, nurse manager's close friendship with employees could make it more difficult to examine the work unit and its functions objectively.

*“There are managers who are very permissive and let the employees behave each in their own way, it is typical that new small managers rise beside them.”*

According to the findings nurse managers sometimes behaved in a manner the employees felt to be unequal.

*“It seems that if you are a strong-willed person, you are more likely to get what you want than a person who is adaptable.” *

**Democratic Leadership Style**

Both nurses and supervisors reported that it was typical for the democratic nurse manager to emphasize teamwork and commitment to work. All employees' participation was important to him/her. The nurse manager worked and discussed work together with the personnel. The employees had a possibility to voice their opinions and take part in problem-solving and decision-making. However, the nurse manager was ultimately expected to be a decision-maker.

*“… and find and make the decision by thinking together and listening to opinions of the employees and discussing together; however, she is in some cases the final decision-maker.”*

There were different perceptions of the nurse managers' positions in this leadership style. On the one hand, they were deemed to be responsible for the work unit and to make reasonable decisions after discussing with the employees. On the other hand, some supervisors felt that some nurse managers did not stand out as managers, but as team members. This meant that the nurse manager's own tasks could be of secondary importance.

*“… she is working a lot with us and she has difficulties performing her own duties as a nurse manager.”*

Supervisors said that a nurse manager had an important role in cooperation and its development with the members of different professional groups and between work units. His/her skills to get the employees to commit to the common goals were deemed as significant. Planning together with the personnel formed a basis for employees' commitment to work. That was essential for the development of the operation of the work unit.

*“… leadership style influences operation as a whole, for example, how a manager gets employees to commit to common decisions”*

**Commanding Leadership Style**

Both nurses and supervisors identified a commanding leadership style, characterized by an emphasis on compliancy and control. Nurses as well as supervisors reported that it was important to the nurse managers with a commanding leadership style to follow clear directions and advice which they expected to get from others, for example, their own superiors. The employees were expected to obey these orders. The nurse manager could ask employees' opinions on how to find a solution to a problem in the work unit; usually s/he had already made a decision and it was not changed by the opinions of the employees. The nurse manager did not think it necessary to explain his/her decisions. The leadership style was described as authoritarian, hierarchical, and inflexible.

*“Nurse managers who do not have the latest knowledge of leadership, they demand that there should be clear rules and laws for everything and there is no flexibility.”*

Commanding leadership style was more common in the 1970s and 1980s and it was now considered traditional and out-of-date. It was, however, described as a convenient leadership style when employees are inexperienced or when there are big changes in the work unit. Nurse managers were described as controlling the behaviour of the personnel, although observations of that kind have diminished considerably.

**Isolating Leadership Style**

Both nurses and supervisors described that nurse managers could isolate themselves from the work unit and retire to their own room where they worked alone without active communication with the employees. In that case the employees felt that they had been left without a leader. Problematic situations like conflicts between employees often arose and they were difficult to repair. Neither the nurse manager nor the employees got the information they needed in their work.

*“The nurse manager is quite isolated, she works alone in her room, we visit her when we have something to discuss with her.”*

#### 3.1.2. Factors Affected by Leadership Style

Both nurses and supervisors reported that nurse manager's leadership style affects employees' job satisfaction and commitment to work. It is felt that nurse manager's fairness and trust in the employees promotes their motivation and participation in work. It is important that the employees have a possibility to develop their professional skills. Leadership style contributes to job satisfaction when the nurse manager has skills to prevent and solve conflicts. 

All the participants reported that nurse manager's skills to lead the work unit and motivate people affect the success of the work unit. Often s/he has to ask for adequate resources. It is important that there are enough trained employees and the employees know and are in charge of their areas of responsibilities. Supervisors remarked on the influence of leadership style on efficiency and economy, because the fluency of operation has an impact on how much money is spent. The nurse manager's influence in developing and changing operation is very important. It is important that the employees have a possibility to take part in development work as well. Nurse manager's leadership style can promote or hinder development in the work unit. 

Supervisors emphasized that nurse managers have a significant role in cooperation within the work unit and outside it. Some nurse managers want to work only inside their own unit, while others take a larger view of the matter. Nurse manager's leadership style has an influence on how externally orientated the staff are and whether they have connections outside the work unit. A nurse manager can promote the continuity of patient care by cooperation with other units. S/he is a role model in how to treat nurse students. 

Both nurses and supervisors felt that problems in the organizational climate, such as conflicts between the employees or dissatisfaction with the nurse leader are reflected in patient care. The activity or passivity of the nurse manager affects the image of the work unit.

*“If there is patient mistreatment, it is the nurse manager whose responsibility it is to decide how to react, for example, “in our unit we treat patients well” or “we do not react at all to this complaint”.”*

All in all, organizational climate, personnel's job satisfaction and commitment, work unit's operation and development work, and cooperation influence the way patient care succeeds and how a patient experiences the care he/she gets. Leadership style has an effect on patient satisfaction and quality of care. If the nurse manager's basic value is good patient care, it influences in many ways his/her leadership style and how s/he organizes things in the work unit. 

### 3.2. Discussion

The discussion is structured around the findings identified above. An isolating leadership style was identified as distinct from the leadership styles that Goleman [23] presented, whereas pace-setting leadership style was not reported. The participants reported that nurse managers used many leadership styles, but normally they had one which they used more than others. Nurses who worked for leaders with resonant leadership styles were more satisfied with supervision and their jobs [34]. Furthermore, visionary leadership style, coaching leadership style, affiliate leadership style, and democratic leadership style seem to promote transformational leadership because they motivate and involve staff. That is why nurse managers should develop themselves in the use of these leadership styles. 

Nurse managers' leadership style depends on many issues, such as organization, situation, and employees. Reynolds and Rogers [35] argue that employees have variable levels of competence depending on the situation. That requires managers to adapt their leadership style. It is important that nurse managers have skills to reflect on their own leadership style and receive feedback about it. That gives them tools to use different leadership styles in different situations.

Health care is meeting ever-increasing new challenges where it has to react rapidly. It is important to the health care organizations to make long-term plans and prepare for the future by paying attention to the needs of inhabitants and the resources needed. The vision is the basis of the goals of the work unit, too. Having knowledge of nursing science and practice gives nurse managers the tools to use a visionary leadership style and make plans for the future. Morjikian et al. [36] argued that communication of future plans, goals, and strategies is important between the nurse manager and the employees. It is important to give information of the vision and explain it regularly to the employees, because sometimes the employees forget the purpose of their work and their working style is not appropriate. When nurse managers work like this they are also carrying out transformational leadership [4, 5]. 

In the future, securing skilled employees will be a big challenge in health care. Vesterinen et al. [29] found that nurse managers with a coaching leadership style appreciated employees' professional skills and encouraged them to study further. Nurse manager's consideration of employees' profession and educational needs influenced nurse retention positively. Kenmore [37] argued that a coaching leadership style works when the employees are keen to develop and make use of possibilities to do so. Education gave employees tools to work and make decisions independently. Although the nurse manager organizes the work unit as a whole and is responsible for the development work in the unit, his/her support has a significant role in helping employees to cope with the problems they meet in their work. This is also an important part in nurse managers' role as emotionally intelligent leaders [10]. 

As a consequence of globalization, both employees and patients come from many different cultures. Their behaviour and habits to express their needs vary. An affiliate leadership style with acceptance of difference could be suited for the multicultural work unit. It is a challenge for the manager to listen sensitively and consider to employees' personal needs individually and at the same time objectively, not forgetting employees' equality. This requires an emotionally intelligent nurse manager [10]. The basis of the leadership style could be supporting the well-being and job satisfaction of the employees. As Kenmore [37] argues, if a nurse manager is too concerned with creating harmony, it can lead to evasion of problems.

Because of shortage of employees, nurses have many possibilities to choose and change their workplaces. Democratic leadership style promoted employees' commitment to work [29]. It is important that the employees can express their opinions and take part in decision-making. 

A commanding leadership style prevents the empowerment of the nurses, because they do not have possibilities to participate in work planning [38]. However, there are situations where a commanding leadership style is appropriate. The majority of Finnish nurses will retire in the next few years and there are many nurses with less work experience in the work units. Employees with less work experience may need clear directions, for example, in acute situations when a patient's life is in danger.

According to Huston [1], essential nurse manager competencies for the future include the ability to create an organization culture that combines high-quality health care and patient/employee safety and highly developed collaborative and team building skills. As a result of this study, an isolating leadership style was found: the nurse manager worked alone without active communication with the employees. The employees have to work without a leader, and that could cause anxiety for the employees who need support from their leader. A good question in this case is who is really leading the work unit. If the leader does not show consideration towards the employees, it could affect their health and well-being negatively [39]. Nurse managers need support to develop the leadership style. Leaders and their supervisors should be considered collectively to understand how leadership influences employee performance [40].

A nurse manager has an important role in leading the work unit as a whole. A work unit is seen as a reflection of the nurse manager. According to Rosengren et al. [41], nurses reported that nursing leadership was considered “being present and available in daily work,” “facilitating professional acknowledgement,” “supporting nursing practice” and “improving care both as a team and as individuals.” A nurse manager with an emotionally intelligent leadership style creates a favourable work climate characterized by innovation, resilience, and change [42]. Nurse managers have to be flexible in the changes they have directly initiated or by which they have been indirectly affected [43]. Leadership style affects the organizational climate and the ways how information is given and communicated and how questions of the day are discussed. The nurse manager creates the basis for how different opinions are handled and problems solved in the work unit.

Nurse manager's leadership style affects the personnel's job satisfaction and commitment. It is perceived that nurse manager's trust in the employees promotes their motivation and participation in work. Way et al. [44] found that trust and job satisfaction are strong links with greater commitment and intent to stay on at work. Nurse managers create basic preconditions for the operation and for development work. 

Leader encourages the employees to develop goals and plan to achieve them. In this way he/she influences the professional development of the personnel [45]. Their skills to build bonds and seek out mutually beneficial relationships affect cooperation in the work unit and around it.

 On the other hand, there is no one and only correct leadership style; the same result can be achieved in many ways. A manager who has the ability to reflect on his/her own behaviour, that is, who has high EI, is better able to regulate and estimate his/her leadership style with different employees in different situations. Leadership style influences patient care and its quality at least indirectly. A nurse manager has a significant role in using a leadership style that promotes good patient care.

## 4. Conclusions

Nurse managers had many leadership styles, but normally they had one that they used more than the others. The nurse managers should consider their leadership style from the point of view of employees, situation factors, and goals of the organization. Leadership styles where employees are seen in a participative, active role have become more common. Together with health care organizations, nursing education programmes should include education of nurse managers to improve their self reflection, through which they are better able to vary their leadership style.
